# Effect of Maternal Marginal Zinc Deficiency on Development, Redox Status, and Gene Expression Related to Oxidation and Apoptosis in an Avian Embryo Model

**DOI:** 10.1155/2021/9013280

**Published:** 2021-10-19

**Authors:** Wei Gao, Liang Huang, Xiufen Zhang, Xinyan Ma, Wence Wang, Yaohui Zheng, Wei Geng, Chuang Liu, Shi Wei, Lin Yang, Yongwen Zhu

**Affiliations:** ^1^Guangdong Provincial Key Laboratory of Animal Nutrition and Regulation, College of Animal Science, South China Agricultural University, Guangzhou 510000, China; ^2^Institute of Animal Science, Guangdong Academy of Agricultural Sciences, Key Laboratory of Animal Nutrition and Feed Science (South China) of Ministry of Agriculture, State Key Laboratory of Livestock and Poultry Breeding, Guangdong Public Laboratory of Animal Breeding and Nutrition, Guangdong Key Laboratory of Animal Breeding and Nutrition, Guangzhou 510640, China; ^3^Waterfowl Division, WENS Group, Yunfu 527300, China

## Abstract

Maternal severe zinc (Zn) deficiency resulted in growth retardation and high mortality during embryonic development in human. Therefore, this study is aimed at evaluating the effect of maternal marginal Zn deficiency on the development and redox status to avoid severe Zn deficiency using an avian model. A total of 324 laying duck breeders at 214 days old were randomly allotted into 3 dietary Zn levels with 6 replicates of 18 ducks per replicate. The birds were fed experimental diets including 3 dietary supplemental Zn levels of 0 mg/kg (maternal Zn-deficient group, 29.2 mg Zn/kg diet), 60 mg/kg (maternal Zn-adequate group), and 120 mg/kg (maternal Zn-high group) for 6 weeks. Dietary Zn levels had on effect on egg production and fertility (*P* > 0.05), whereas dietary Zn deficiency decreased breeder plasma Zn concentration and erythrocytic alkaline phosphatase activity at week 6 and inhibited erythrocytic 5′-nucleotidase (5′-NT) activity at weeks 2, 4, and 6 (*P* < 0.05), indicating that marginal Zn-deficient status occurred after Zn depletion. Maternal marginal Zn deficiency increased embryonic mortality and contents of superoxide anion radical, MDA, and PPC and reduced MT content and CuZnSOD activity in duck embryonic livers on E29. The MDA content was positively correlated with embryonic mortality. Maternal marginal Zn deficiency increased *BCL2-associated X protein* and *Caspase-9* mRNA expressions as well as decreased *B-cell lymphoma-2* and *MT1* mRNA and signal AKT1 and ERK1 protein expressions (*P* < 0.05). Breeder plasma Zn concentration and erythrocytic 5′-NT activities at week 6 were positively correlated with GSH-Px activity and *GPx*, *MT1*, and *BCL2* mRNA expressions in embryonic livers on E29. In conclusion, erythrocytic 5′-NT activity could be more rapid and reliable to monitor marginal Zn-deficient status. Marginal Zn deficiency impaired hatchability and antioxidant defense system and then induced oxidative damage and apoptosis in the embryonic liver, contributing to the greater loss of duck embryonic death.

## 1. Introduction

Zinc (Zn) is an essential trace mineral required for maintaining the normal growth and development of embryos [1]. Maternal marginal Zn deficiency could lead to the susceptibility of embryonic death predominantly [2]. Furthermore, severe Zn deficiency in maternal diets resulted in growth retardation, abnormal development, and increased mortality of embryos [3]. Therefore, it is crucial to assess or predict maternal marginal Zn nutritional status to prevent embryos subjected to severe Zn deficiency. Some traditional and reliable biochemical or functional indicators (e.g., tissue Zn contents and bone mineralization) have been proposed to estimate maternal Zn status [4]. Only when Zn deficiency is relatively severe is it possible to detect changes in tissue Zn concentrations. It is necessary to select some specific sensitive biomarkers to predict maternal marginal Zn status. Some studies in rats and humans revealed that plasma Zn concentration could be used as a sensitive biomarker in response to Zn status [5, 6]. Alkaline phosphatase (ALP) is a Zn metalloenzyme, and its activity in blood was decreased by 80% when the Zn content was reduced from 96 mg/kg to 1.2 mg/kg in the rat [7]. The 5′-nucleotidase (5′-NT) activity, like a cell membrane enzyme in erythrocyte and thymulin [8], was more sensitive to mild Zn deficiency than plasma Zn concentration [9, 10]. It is speculated that the activities of ALP and 5′-NT could be developed as specific sensitive biomarkers for predicting the marginal Zn-deficient status.

Maternal inadequate Zn supply decreased Zn deposition in egg yolk and then reduced the Zn mobilization from storage sites to the tissues of the developing embryos [11]. Zn as a cofactor of some distinct metalloenzymes, such as metallothioneins (MTs) and copper-zinc superoxide dismutase (CuZnSOD), has a diverse range of biological reactions for maintaining embryonic development [12, 13]. Marginal Zn deficiency in maternal diet could induce some adverse effects on antioxidant ability and antiapoptosis during embryonic development [14]. In vivo studies revealed that marginal Zn deficiency throughout gestation caused induction of oxidative stress and impaired the normal development of the fetal brain in the rat [15]. Maternal dietary Zn supplementation could effectively eliminate chick embryonic mortality induced by maternal hyperthermia via enhancing antioxidant ability [12]. In vitro studies also have demonstrated that Zn deficiency in cell culture was conducive to the production of reactive oxygen (ROS) and caspase activation [16]. In addition, Zn deficiency induced apoptosis involving the inhibition of growth factor signaling pathways during embryonic and fetal development [2]. It is hypothesized that a deficient in Zn availability could cause alterations in redox status and then lead to oxidative damage and cell apoptosis in tissues, contributing to abnormal embryo development. In the current study, effect of maternal marginal Zn deficiency on embryonic development, redox status, and gene expressions related to antioxidant and antiapoptosis abilities were studied in an avian embryo model.

## 2. Methods and Materials

### 2.1. Animals and Diets

All animal protocols used in the present study were approved by the South China Agricultural University Institutional Animal Care and Use Committee. A total of 370 186-day-old Muscovy duck breeders were obtained from a commercial duck breeder farm (WENS Group, Yunfu, Guangdong, China) and housed in the caged system for 4-week adaptation period. During the adaptation period, welfare-related assessments and interventions were carried out to meet the requirements of the South China Agricultural University Institutional Animal Care and Use Committee. All breeder ducks were fed restrictively (160 g/d/bird) with a commercial feed at the nutritional level (11.32 MJ metabolizable energy/kg, 180 g crude protein/kg, 7.0 g lysine/kg, 7.2 g methionine+cysteine/kg, 24.0 g calcium/kg, 3.8 g available phosphorus/kg, and 40 mg Zn/kg) during adaptation period. Then, 324 laying duck breeders aged 214 days were selected, balanced for laying rate, and then randomly allotted into 3 dietary Zn levels with 6 replicates of 18 ducks per replicate. The experimental period lasted for 6 weeks. The experimental diets included 3 dietary supplemental Zn levels of 0 mg/kg (maternal Zn-deficient group, MZD), 60 mg/kg (maternal Zn-adequate group, MZA), and 120 mg/kg (maternal Zn-high group, MZH) as inorganic Zn sulfate. The diets were formulated to meet or exceed the nutritional requirements of laying duck breeders according to the national agricultural industry standard of China (NY/T 2122-2012). According to the experimental treatments, single batch of basal diet was mixed and then divided into 3 aliquots with or without supplementation of Zn sulfate (10024018, Sinopharm Chemical Reagent Co., Ltd., Beijing, China). The composition of the basal diet is shown in Table 1. The analyzed values of Zn contents in MZD, MZA, and MZH diets were 29.2, 87.4, and 163.4 mg/kg, respectively. All birds had diet restrictions (160 g/d/bird) and access to water *ad libitum*. The breeders received 16 h of daily lighting from 04 : 30 am to 08 : 30 pm. Room temperature and humidity were controlled by the air-conditioner and recorded daily. Manure was removed through an automatic belt system daily. All male duck breeders were fed the same diet formulated to meet the nutritional requirements throughout the experimental period. The practice of semen collection started at 175 days of age, and the quality of semen was determined by the volume and numbers of semen and sperm motility. During the experimental feeding period, semen was collected and mixed from male duck breeders from 182 to 224 days of age. Artificial insemination was performed every four days. At the end of the experiment, all breeders were removed and were fed restrictively with a commercial feed to meet the nutrient requirements of birds. Lighting and feeding management were performed according to the instructions of Muscovy duck male breeder management guidelines. At the ending of feeding trial, the recovery performance standards and normal behaviors were done to evaluate the optimum welfare of the rest of breeders weekly.

During the 6-week experimental period, all eggs were collected from each replicate and recorded daily. Feed consumption and egg weight were measured weekly. Feed intake was calculated by dividing the total feed consumed by the number of ducks per replicate per day. The eggs were collected during the last week of the experimental period and then were stored in one storage room at a temperature of 15°C and a relative humidity of 70%. At the end of storage, all the eggs from one replicate (approximately 100 eggs) of the 3 dietary treatments were placed on the same egg tray (6 trays total) and then incubated in the same incubator (9TDJ-A, LanTianJiao Electronic Technology Company, Beijing, China). The eggs were incubated at a temperature of 37.5 ± 0.5°C and relative humidity of 55 ± 5% until E30 and then were transferred to hatchers. Eggs were candled on E7 and E28 to identify infertile, cracked, or nonviable embryos. All removed eggs on E7 and E28 were counted, opened, and visually evaluated also to determine the actual embryonic mortality. Fertility was expressed as the percentage of fertile eggs in the total number of eggs set for each replicate per treatment. Hatchability and embryonic mortality were expressed as percentages of the hatched birds and dead embryos in the total number of fertile eggs of each replicate per treatment, respectively.

### 2.2. Sample Collections

The feed ingredients and diet samples from all the treatments were collected and analyzed for crude protein, calcium, and Zn contents. After fasting for 12 hours, blood samples were collected via a bronchial vein from the same two duck breeders in each replicate on the last day of weeks 2, 4, and 6 during the experimental period, respectively. Blood samples were separated into plasma and erythrocytes by centrifugation at 3000 × g for 15 min at 4°C. Erythrocyte samples were washed three times in cold isotonic saline (0.9%, v/w) and then haemolyzed with a ninefold volume of phosphate buffer (pH 7.4). The equal volume of plasma and haemolyzed erythrocytes were pooled and stored at -20°C for further analysis.

Twelve eggs from each treatment (2 per replicate) were collected on the last day of week 6 of experimental period. The separated yolk from 2 eggs per replicate was pooled together and stored at -20°C for Zn analysis. On E29, 24 embryos (4 per replicate) from each treatment were killed by cervical dislocation. Equal weight subsamples of the livers from the 4 embryos in each replicate were pooled into one sample for analysis. Total one-gram liver sample of each replicate was homogenized at 8000 × g for 10 seconds in 9 mL of 0.9% sodium chloride buffer on ice and centrifuged at 3000 × g at 4°C for 15 min, and the resultant supernatant was used for the analyses of antioxidant ability. The liver samples from the embryos were immediately dissected and frozen in liquid nitrogen and then stored at -80°C for further investigation of the gene and protein expressions.

### 2.3. Determination of Zn Concentration

Zinc contents in samples including diets, breeder plasma, and egg yolk were measured using an inductively coupled plasma emission spectroscope (IRIS Intrepid II, Thermal Jarrell Ash, Waltham, MA) after wet digestions with HNO_3_ and HClO_4_ as described by Zhu et al. [11]. Validation of the mineral analysis was conducted using bovine liver powder (GBW (E) 080193, National Institute of Standards and Technology, Beijing, China) as a standard reference material. Calibrations for the Zn assay were conducted with a series of mixtures containing graded concentrations of standard solutions of Zn.

### 2.4. Determination of Zn Metalloenzyme Activities in Breeder Erythrocytes

ALP activity was measured using a HITACHI 7180 automatic biochemical analyzer (Hitachi Ltd., Tokyo, Japan) with a detection kit (A059-2-2, Nanjing Jiancheng Bioengineering Institute). CuZnSOD activity was determined by subtracting manganese superoxide dismutase (MnSOD) activity from total SOD (TSOD) activity according to the nitrite method [17]. 5′-NT activity was assayed by the determination of the P_i_ liberated from the substrate nucleotide as described previously [18]. Total protein concentration in erythrocytes was determined using a BCA Protein Assay Kit (23225, Pierce). All indices of erythrocytes were expressed as nitrite units per milligram protein.

### 2.5. Determination of Indices Related to Oxidative Damage

The activity of superoxide anion radical production was calculated and expressed as a percentage of control (vitamin C) based on the inhibition rate of superoxide anion radicals from the xanthine and xanthine oxygenase reaction following the instruction of a commercial assay (A052-1-1, Nanjing Jiancheng Institute of Bioengineering). The malondialdehyde (MDA) and protein carbonyl content (PCC) were determined by thiobarbituric acid colorimetric (A003, Nanjing Jiancheng Institute of Bioengineering) and 2,4-dinitrophenylhydrazine methods according to kits (A087, Nanjing Jiancheng Institute of Bioengineering), respectively. The 8-hydroxy-2-deoxyguanosine (8-OHdG) was determined with a commercially available ELISA test kit (H165, Nanjing Jiancheng Institute of Bioengineering). All indices of supernatant were expressed as nitrite units per milligram protein.

### 2.6. Determination of Antioxidant Enzyme Activities

Supernatant of the liver homogenization solution was used to measure the activities of glutathione peroxidase (GSH-Px) and catalase (CAT) using the commercial kits (A005-1-2 and A007-1-1, Nanjing Jiancheng Bioengineering Institute) according to the instructions of the manufacturer. The total SOD (TSOD) and MnSOD activities were measured following the nitrite method described by Zhu et al., and CuZnSOD activity was calculated by subtracting MnSOD activity from TSOD activity. MT content was determined using an ELISA kit for duck species (CG3309, Waltham).

### 2.7. RT-qPCR for Gene mRNA Expression

Total RNA was extracted from the embryonic liver tissues using Trizol reagent (15596018, Life Technologies), and then, reverse-transcription was performed using QuantiTech Reverse Transcription Kit (205311, Qiagen) following the manufacturer's protocols with genomic DNA wiping off. The protocol of two-step PCR using ABI Power SYBR Green PCR Master Mix was conducted as described previously [17]. The primer sequences are listed in Table S1. The *glyceraldehyde 3-phosphate dehydrogenase* (*GAPDH*) was used to normalize the expressions of the targeted genes. The 2^−△△Ct^ was used to calculate the mRNA level of each target gene using the MZD group as the reference group.

### 2.8. Western Blotting for Protein Expression

Total protein was extracted with ice-cold RIPA lysis buffer (P0013B, Beyotime Institute of Biotechnology). The procedure following the preparation of the protein sample and SDS-PAGE, blotting transfer, and detection of the protein-specific antibodies were performed as described previously [12]. The primary antibodies are listed in Table S2.

### 2.9. Statistical Analyses

All data were analyzed by one-way ANOVA using the PROC GLM procedure of the SAS (SAS Inst. Inc., Cary, NC). Additionally, the significant effect of dietary Zn on breeder plasma Zn concentration and erythrocytic Zn metalloenzyme activities was analyzed for each sampling time. All data were presented as mean ± SEM. The cage served as the experimental unit for the indices of reproductive performance, while the pooled sample within a cage served as the experimental unit for other indices. Differences among means were tested by the Fisher's Least Significance Difference test method, and statistical significance was set at *P* ≤ 0.05. The correlations of the parameters between the stages of breeder at week 6 during experimental period and embryo on E29 were performed by Pearson correlation coefficients.

## 3. Results

### 3.1. Productive Performance and Zn Concentration in Egg Yolk

**Figure 1:**
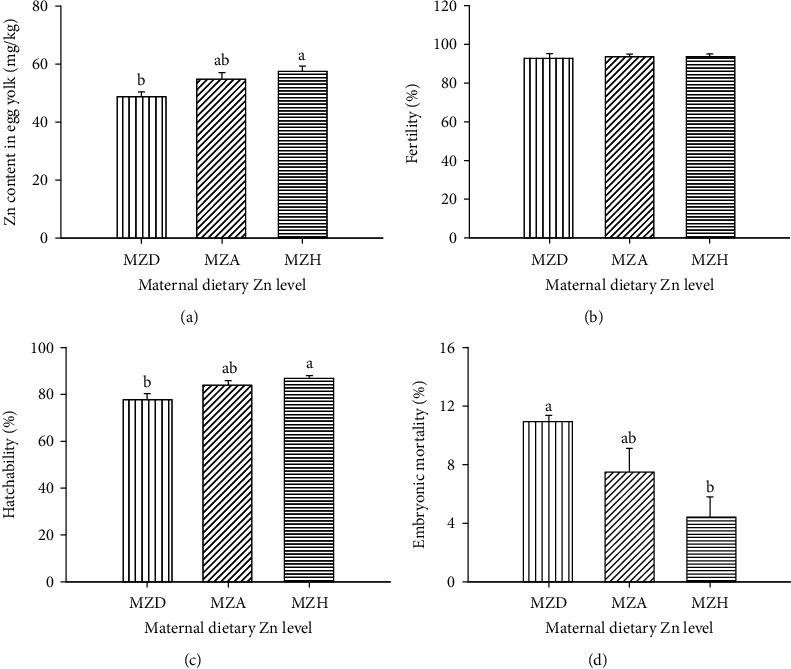
Effect of maternal dietary Zn on Zn concentration in (a) egg yolk, (b) fertility, (c) hatchability, and (d) embryonic mortality. The Zn content in egg yolk was measured on a fresh basis. All values are expressed as means ± SE. Means with different letters (a and b) differ significantly (*P* < 0.05). Mean represented the average value of 6 replicates (*n* = 6). MZD: maternal Zn-deficient group (0 mg Zn/kg diet); MZA: maternal Zn-adequate group (60 mg Zn/kg diet); MZH: maternal Zn-high group (120 mg Zn/kg diet).

Dietary Zn levels did not affect (*P* > 0.05) egg weight (MZD 75.3 vs. MZA 76.1 vs. MZH 76.1 g), laying rate (MZD 83.9% vs. MZA 84.0% vs. MZH 83.2%), egg mass (MZD 63.2 vs. MZA 63.9 vs. MZH 63.3 g/bird/day), and feed: egg ratio (MZD 2.48 vs. MZA 2.44 vs. MZH 2.47) of duck breeders (Fig. S1). Maternal dietary Zn levels affected (*P* < 0.05) Zn concentration in egg yolk (Figure 1(a)), hatchability (Figure 1(c)), and embryonic mortality (Figure 1(d)), but did not influence fertility (*P* > 0.05, Figure 1(b)). The MZD group had a lower Zn content in egg yolk and hatchability as well as higher embryonic mortality than the MZH group, with no differences between MZD and MZA groups.

### 3.2. Plasma Zn Concentration and Metalloenzyme Activities in Erythrocytes

**Figure 2:**
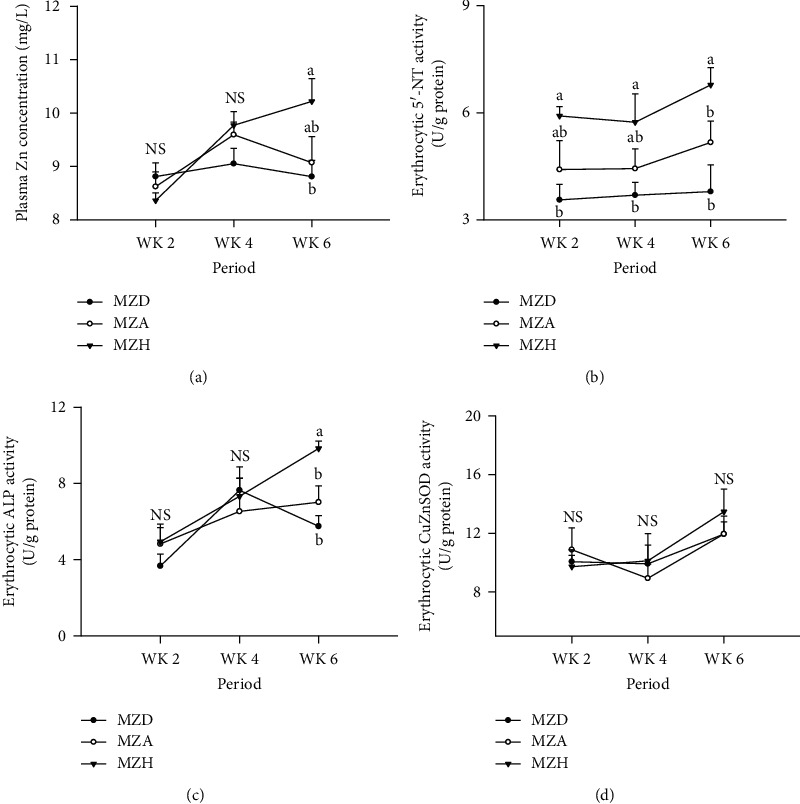
Effects of dietary Zn and age on (a) plasma Zn concentration and (b) erythrocytic 5′-NT, (c) ALP, and (d) CuZnSOD activities of duck breeders at weeks 2, 4, and 6 during experimental period. All values are expressed as means ± SE. Means with different letters (a and b) differ significantly (*P* < 0.05) among dietary Zn groups at weeks 2, 4, and 6, respectively. Means with the letter (NS) showed no significant differences (*P* > 0.05) among dietary Zn groups at weeks 2, 4, and 6, respectively. Mean represented the average value of 6 replicates (*n* = 6). MZD: maternal marginal Zn-deficient group (0 mg Zn/kg diet); MZA: maternal Zn-adequate group (60 mg Zn/kg diet); MZH: maternal Zn-high group (120 mg Zn/kg diet); CuZnSOD: copper-zinc superoxide dismutase; ALP: alkaline phosphatase; 5′-NT: 5′-nucleotidase; WK: week; NS: no significant differences.

Dietary Zn levels had no effect on CuZnSOD activity in erythrocytes of duck breeders at weeks 2, 4, and 6 (*P* > 0.05, Figure 2(a)). Compared to MZH, MZD decreased erythrocytic 5′-NT activity at weeks 2, 4, and 6 and did not differ from MZA at 2 and 4 weeks of age as well as neither MZA differed from MZH at weeks 2, 4, and 6. MZD decreased plasma Zn concentration and erythrocytic ALP activity of breeders at week 6, but did not affect those at weeks 2 and 4.

### 3.3. Oxidative Damage and Antioxidant Enzyme Activities in Embryonic Livers

**Figure 3:**
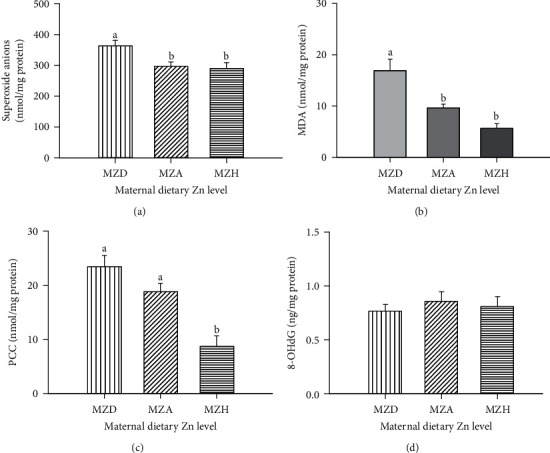
Effect of maternal dietary Zn on the contents of (a) superoxide anion radical, (b) MDA, (c) PCC, and (d) 8-OHdG in embryonic livers on E29. All values are expressed as means ± SE. Means with different letters (a and b) differ significantly (*P* < 0.05). Mean represented the average value of 6 replicates (*n* = 6). MZD: maternal Zn-deficient group (0 mg Zn/kg diet); MZA: maternal Zn-adequate group (60 mg Zn/kg diet); MZH: maternal Zn-high group (120 mg Zn/kg diet); MDA: malondialdehyde; PCC: protein carbonyl content; 8-OHdG: 8-hydroxy-2-deoxyguanosine.

**Figure 4:**
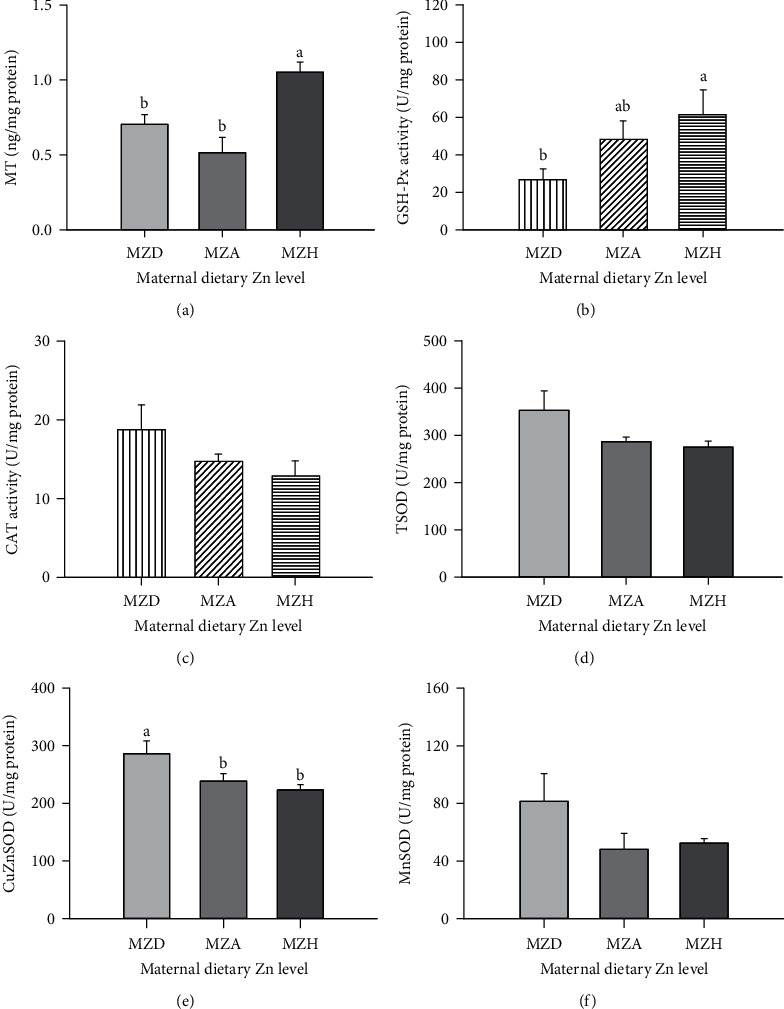
Effect of maternal dietary Zn on (a) MT content and (b) GSH-Px, (c) CAT, (d) TSOD, (e) CuZnSOD, and (f) MnSOD activities in embryonic livers at E29. All values are expressed as means ± SE. Means with different letters (a–c) differ significantly (*P* < 0.05). Mean represented the average value of 6 replicates (*n* = 6). MZD: maternal Zn-deficient group (0 mg Zn/kg diet); MZA: maternal Zn-adequate group (60 mg Zn/kg diet); MZH: maternal Zn-high group (120 mg Zn/kg diet); MT: metallothionein; GSH-Px: glutathione peroxidase; CAT: catalase; TSOD: total superoxide dismutase; CuZnSOD: copper-zinc superoxide dismutase; MnSOD: manganese superoxide dismutase.

Maternal dietary Zn levels influenced (*P* < 0.05) the contents of superoxide anion radical, MDA, PCC (Figures 3(a)–3(c)), and MT (Figure 4(a)) and activities of GSH-Px (Figure 4(b)) and CuZnSOD (Figure 4(e)), but did not affect (*P* > 0.05) on the 8-OHdG content (Figure 3(d)) and CAT activity (Figure 4(c)) in embryonic liver on E29. Maternal dietary Zn deficiency increased the superoxide anion radical, MDA, and PPC contents and decreased GSH-Px activity in embryonic livers. The MT content was lower, and CuZnSOD activity was higher in embryonic liver in MZD than in MZH groups. There were no differences in MT content between MZD and MZA groups as well as CuZnSOD activity between MZA and MZH groups.

### 3.4. Target Gene and Protein Expressions in Embryonic Livers

**Figure 5:**
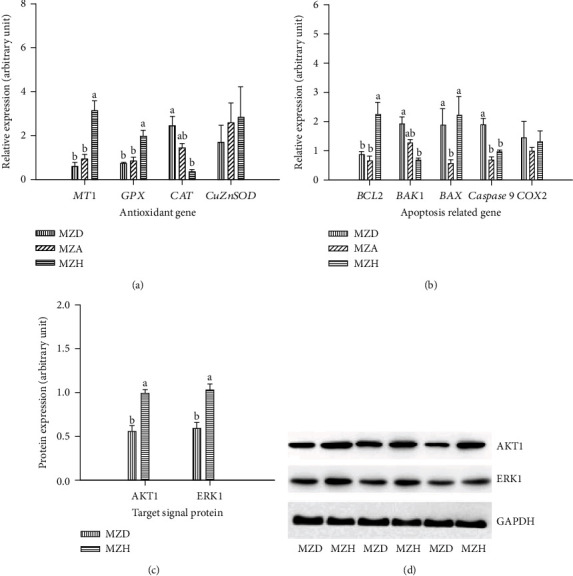
Effects of maternal dietary Zn levels on of (a, b) antioxidant genes (*MT1*, *GPx*, *CAT*, and *CuZnSOD*) and antiapoptotic gene (*BCL2*, *BAK1*, *BAX*, *Caspase*-*9*, and *COX2*) mRNA expressions as well as (c) signaling AKT1 and ERK1 protein expressions in the embryonic liver. (d) Representative immunoblots of the indicated proteins were listed. The *GAPDH* mRNA expression was used to normalize the expressions of the targeted genes. The GAPDH protein expression was selected to normalize target protein expressions. Means with different letters (a and b) differ significantly (*P* < 0.05). Mean represented the average value of 6 replicates (*n* = 6). MZD: maternal Zn-deficient group (0 mg Zn/kg diet); MZA: maternal Zn-adequate group (60 mg Zn/kg diet); MZH: maternal Zn-high group (120 mg Zn/kg diet); *MT1*: *metallothionein 1*; *GPx*: *glutathione peroxidase*; *CAT*: *catalase*; *CuZnSOD*: *copper-zinc superoxide dismutase*; *BCL2*: *B-cell lymphoma-2*; *BAK1*: *BCL2 antagonist/killer 1*; *BAX*: *BCL2-associated X protein*; *COX2*: *cyclooxygenase-2*.

As shown in Figure 5, embryonic liver had higher *MT1* and *BCL2* mRNA expression and lower *CAT*, *BAK1*, and *Caspase*-*9* mRNA expression in the MZH group than in the MZA group (*P* < 0.05), whereas there were no differences in these indexes between MZD and MZA groups. MZA group had lower *BAX* mRNA expression in embryonic liver than those from MZD and MZH groups (*P* < 0.05), with no differences between MZD and MZH groups. Compared to the MZD group, the MZH group had higher AKT1 and ERK1 protein expression of in embryonic liver (*P* < 0.05).

### 3.5. Correlation of Some Measured Parameters between Breeders and Embryos

As shown in Table 2, plasma Zn concentration was positively (*P* < 0.01) correlated with erythrocytic ALP and 5′-NT activities in breeders at week 6. Breeder plasma Zn concentration and erythrocytic ALP and 5′-NT activities were positively correlated with GSH-Px activity as well as *GPx*, *MT1*, and *BCL2* mRNA expression in embryonic liver on E29 (*P* < 0.05). The MDA content had positive correlation with embryonic mortality (*P* < 0.01), while the MDA content had negative correlation with GSH-Px activity and *MT1* mRNA expression in the embryonic liver (*P* < 0.05).

## 4. Discussion

The development and growth of poultry embryos are dependent upon the nutrient deposits in the eggs [19]. The yolk sac provides the chicken embryo with essential mineral nutrients for embryonic growth [20, 21]. Zinc as an essential nutrient is required in small amounts for normal growth and development of the avian embryo functioning as catalytic or structural cofactors in metal-containing enzymes [1]. Previous studies demonstrated that supplemental Zn in diets was essential to achieve normal reproductive performance in chickens [1] and rats [16]. However, the results from our study indicated that adding Zn to the diets had no effects on the characteristics of egg production performance of duck breeders, which was inconsistent with the positive results reported by laying hens [22]. The discrepancy between the studies may depend on the differences in the genetic breeds (Muscovy duck breeder vs. Hisex Brown laying hen), supplemental Zn sources (Zn sulfate vs. Zn oxide), and Zn depletion periods (6 weeks vs. 12 weeks) of the birds. However, feeding Zn deficiency in duck breeder diets resulted in a lower hatchability due to an increase in the average day of embryonic mortality. The above results indicated that Zn requirement for laying performance might not be sufficient to maintain the hatchability and embryonic development of laying ducks, suggesting that embryonic development was much more sensitive to maternal marginal Zn deficiency than egg production. However, severe Zn deficiency in hen diets could impair both egg production and embryonic development [23], whereas maternal Zn supplementation or *in ovo* Zn injection in the yolk can eliminate these adverse effects [24]. Therefore, it is necessary to assess Zn status by measuring some specific sensitive indicators to prevent the marginal or severe Zn deficiency in breeder diets.

Some traditional and reliable biochemical or functional indicators (e.g., tissue Zn contents and bone mineralization) have been proposed for estimating Zn status in poultry breeders [25]. In fact, only when Zn deficiency is relatively severe is it possible to detect changes in Zn concentrations in tissues. Some studies in rats and humans revealed that plasma Zn concentration and Zn metalloenzyme activities could be used as sensitive biomarkers to permit estimation of the prevalence of marginal Zn deficiency [4]. For example, plasma Zn concentration is approximately 50 times lower than that in tissues, and slight differences in uptake or release of Zn from these peripheral sites could profoundly affect the plasma Zn concentration [4]. Studies in the pregnancy of rats and humans also found a significant increase in the plasma Zn concentrations following supplementation [5, 6]. In this study, dietary Zn deficiency decreased the plasma Zn concentration of duck breeders at week 6 compared to other two groups, but did not occur at weeks 2 and 4. A similar change tendency responded to dietary Zn level was observed in erythrocytic ALP activity. Dietary Zn deficiency decreased erythrocytic 5′-NT activity at weeks 2, 4, and 6. The positive correlations between plasma Zn concentration and erythrocytic ALP and 5′-NT activities at week 6 implied that marginal Zn-deficient status occurred with the prolonged dietary Zn depletion. Moreover, erythrocytic 5′-NT activity responded to the more pronounced Zn-deficient status was more rapidly and reliably and consequently possessed the capacity to prevent the possible deleterious effects of severe Zn deficiency.

Maternal inadequate Zn decreased Zn deposition in the yolk, implied that Zn supply to target tissues of the developing embryos could decline. The liver is the most important organ for the storage and homeostatic regulation of Zn metabolism in the avian embryo [26]. Zinc as a cofactor of some distinct metalloenzymes [27], such as MT and CuZnSOD, was thought to be particularly important for maintaining Zn-dependent functions of antioxidant ability during chick embryonic development [12]. Previous studies have been demonstrated that severe Zn deficiency in maternal diets resulted in growth retardation, abnormal development, and increased mortality of embryos [3, 11]. In the current study, the effect of maternal marginal Zn deficiency on embryonic development was studied. Maternal marginal Zn deficiency decreased the ability to scavenge superoxide anion radical production in association with the increased MDA and PPC contents. Previous studies have reported that the excessive ROS from oxidative stress led to the damage of lipid and protein and then could arrest the development of embryos in human [28]. Compared to the maternal high Zn group, the maternal marginally Zn-deficient group decreased the MT content in livers of duck embryos. Similar findings were reported for the developing chick embryo showing the consistency between hepatic Zn levels and redox [26]. The positive correlation between MDA content and embryonic mortality also implied that the impaired antioxidant defense system induced by marginal Zn deficiency could contribute to more significant loss of the embryos. Furthermore, the parallel reduced *MT1* mRNA expression was observed in embryonic livers from breeder fed a maternal marginal Zn deficient diet. Studies have demonstrated that MT expressions correlated with hepatic Zn accumulation during development could protect against the oxidative damage of Zn deficiency during pregnancy in transgenic and knockout mice [29, 30]. In chicken [12] and mouse [31], it is proved that maternal Zn deficiency suppressed *MT* mRNA expression of offspring embryos via epigenetic regulation by the DNA hypermethylation and histone hypoacetylation of the gene promoter. Zn deficiency has been shown to initiate apoptosis during development, altering embryogenesis [16]. In our study, the *BAK1*, *BAX*, *and Caspase*-*9* mRNA expressions in related to cell apoptosis were increased in the liver of marginal Zn-deficient duck embryos. Recent reports have been shown that the enhanced MT expressions presented more excellent antiapoptotic effects *in vivo* [32] and *in vitro* [33], which was confirmed in marginal Zn-deficient embryos in the present study. Maternal dietary Zn deficiency decreased antiapoptotic gene *BCL2* mRNA expression in the embryonic liver to promote apoptosis. The negative correlation between MDA content and embryonic *MT1* and *BCL2* mRNA expressions indicated that maternal marginal Zn deficiency might induce cell apoptosis due to the oxidative damage. In addition, a decrease of the AKT1 and ERK1 protein levels in marginal Zn-deficient embryos suggests that Zn deficiency-induced apoptosis could be involved in growth factor signaling of AKT and ERK pathways by inhibiting cell cycle machinery [2].

## 5. Conclusions

In the present study, breeder erythrocytic 5′-NT activity could be developed as a sensitive biomarker to rapidly and reliably monitor the marginal and more pronounced Zn-deficient status. Maternal Zn deficiency impaired hatchability and increased embryonic mortality of duck embryos, which was positively correlated with embryonic liver MDA content. Maternal marginal Zn deficiency impaired antioxidant defense system and induced oxidative damage and apoptosis in embryonic liver. These deleterious effects possibly contributed to the greater loss of embryos during the developing stage.

## Figures and Tables

**Table 1:** Composition and nutrients levels of the basal diets for laying duck breeders during the experimental period (as-fed basis).

Item (%)	Laying period
Corn	51.67
Soybean meal	17.70
Corn gluten meal	7.75
Wheat middlings	8.97
Lard	1.84
Dicalcium phosphate	1.80
Limestone	8.50
Sodium chloride	0.30
DL-methionine	0.27
L-Lysine·HCl	0.20
Vitamin and mineral premix^1^	1.00
Total	100
Nutrient composition	
Calculated value (%)	
Metabolizable energy (MJ/kg)	11.63
Crude protein^2^	18.51
Calcium^2^	3.70
Total phosphate	0.60
Nonphytin phosphorus	0.44
Lysine	0.91
Methionine	0.57
Methionine+cysteine	0.84
Zinc^2^	29.2

^1^Provided per kilogram of diet without Zn addition: vitamin A, 5,000 IU; vitamin D_3_, 800 IU; vitamin E, 20 IU; thiamine, 2.0 mg; riboflavin, 15 mg; pyridoxine, 4.0 mg; vitamin B_12_, 0.02 mg; calcium pantothenate, 10 mg; folate, 0.15 mg; niacin, 60 mg; biotin, 0.20 mg; choline (choline chloride), 1,500 mg; Cu (CuSO_4_·5H_2_O), 8 mg; Fe (FeSO_4_·7H_2_O), 80 mg; Mn (MnSO_4_·H_2_O), 100 mg; Se (NaSeO_3_), 0.3 mg; and I (KI), 0.4 mg. ^2^Analysed values based on triplicate determinations.

**Table 2:** Correlation between the measured parameters of Zn supply in the breeders and embryos.

Item	Breeder stage^1^				Embryonic stage^2^					
	Embryonic mortality	Plasma Zn concentration	Erythrocytic ALP activity	Erythrocytic 5′-NT activity	MDA content	MT content	GSH-Px activity	*GPx* mRNA	*MT1* mRNA	*BCL2* mRNA
Embryonic mortality	1.00									
Plasma Zn concentration	-0.30	1.00								
Erythrocytic ALP activity	-0.42	0.71^∗∗^	1.00							
Erythrocytic 5′-NT activity	-0.50	0.75^∗∗^	0.61^∗^	1.00						
MDA content	0.81^∗∗^	-0.55^∗^	-0.36	-0.54^∗^	1.00					
MT content	-0.50	0.26	0.63^∗∗^	0.39	-0.36	1.00				
GSH-Px activity	-0.45	0.80^∗∗^	0.60^∗^	0.68^∗∗^	-0.58^∗^	0.22	1.00			
*GPx* mRNA	-0.39	0.63^∗∗^	0.54^∗^	0.69^∗∗^	-0.43	0.44	0.70^∗∗^	1.00		
*MT1* mRNA	-0.40	0.75^∗∗^	0.70^∗∗^	0.65^∗∗^	-0.49^∗^	0.47^∗^	0.74^∗∗^	0.88^∗∗^	1.00	
*BCL2* mRNA	-0.37	0.60^∗^	0.49	0.68^∗∗^	-0.40	0.54^∗^	0.67^∗∗^	0.92^∗∗^	0.87^∗∗^	1.00

^1^Embryonic mortality, plasma Zn concentration, erythrocytic ALP, and 5′-NT activity were measured in breeders at week 6 during experimental period. ^2^The MDA and MT contents, GSH-Px activity, and target gene mRNA expressions were determined in embryonic livers on E29. ALP: alkaline phosphatase; 5′-NT: 5′-nucleotidase; MDA: malondialdehyde; GSH-Px: glutathione peroxidase; *GPx*: *glutathione peroxidase*; *MT1*: *metallothionein 1*; *BCL2*: *B-cell lymphoma-2.*^∗^*P* < 0.05; ^∗∗^*P* < 0.01.

## Data Availability

The data used to support the findings of this study are included within the article.

## Supplementary Materials

Supplementary Materials The data used to support the findings of this study are included within the article. Fig. S1: effect of dietary Zn on egg weight, laying rate, egg production, feed intake, and feed/egg ratio. Supplemental Table 1: nucleotide sequences of specific primers for RT-qPCR. Supplemental Table 2: summary of the antibodies used for western blotting.
