# Supplementary material for: Effect of Maternal Marginal Zinc Deficiency on Development, Redox Status, and Gene Expression Related to Oxidation and Apoptosis in an Avian Embryo Model
Source: Oxid Med Cell Longev. 2021 Oct 19;2021:9013280. doi: 10.1155/2021/9013280 (PMC8548136; doi:10.1155/2021/9013280)
Supplement: Supplementary Materials — The data used to support the findings of this study are included within the article. Fig. S1: effect of dietary Zn on egg weight, laying rate, egg production, feed intake, and feed/egg ratio. Supplemental Table 1: nucleotide sequences of specific primers for RT-qPCR. Supplemental Table 2: summary of the antibodies used for western blotting. [file 9013280.f1.docx]

**Supplementary information**

**Table S1** Nucleotide sequences of specific primers for RT-qPCR

| Target gene | Gene bank accession no. | Sequence (5'-3') |
| --- | --- | --- |
| *GAPDH* | XM_005016745.1 | F：GGTGCTAAGCGTGTCATCATCTC  R：CCCCCTCAGCTGATGCTCCCATGA |
| *CuZnSOD* | XM_005019265.4 | F: CCTGTGGTGTCATCGGAATA  R: TTGAACGAGGAAGAGCAAGTA |
| *GPx* | XM_027467953.1 | F: CAGTACATCATCTGGTCGCC  R: CCTGGATCTTGATGGTTTCG |
| *CAT* | XM_027458335.1 | F: CTGTTGAGGAAGCAGGAAGG  R: GAAAGACCAGGATGGGTAGTTG |
| *BCL2* | XM_005028719.1 | F: TCCTCTCTCCCTTCCTCTTGCT  R: CTCCCTCTCGGAAATCATGGCTCATCC |
| *Bax* | KY788660.1 | F: CATCAAGGCTCTGTTCTCGCA  R: AAGATGGTGAGGGTCTGCC |
| *BAK1* | XM_005026829.1 | F: TTGCAGCTCACCAAGGAGAA  R: GATTACTTCATCAAGATTGCCTCCAGCC |
| *COX2* | NM_001167718.1 | F: TCCACCAACAGTGAAGGACA  R: GGACCAAGCCAAACACCTC |
| *Caspase 9* | XM_013095294.1 | F: GCTGCTTCAACTTCCTCCGTAA  R: CATCTCCACGGACAGACAAAGG |

*CuZnSOD* = *copper-zinc superoxide dismutase*; *GPx* = *glutathione peroxidase*; *CAT* = *catalase*; *BCL2* = *B-cell lymphoma-2*; *BAK1* = *BCL2 antagonist/killer 1*; *BAX* = *BCL2-associated X protein*; *COX2* = *cyclooxygenase-2*; F = forward; R= reverse.

**Table S2** Summary of the antibodies used for western blotting

| Antibodies | Molecular weight (KD) | Host species | Source | Clonality | Catalogue no. | Dilution |
| --- | --- | --- | --- | --- | --- | --- |
| GAPDH | 37 | Rabbit | Abcam | Polyclonal | ab22555 | 1:5000 |
| AKT1 | 56 | Rabbit | GeneTek | Polyclonal | GTX128415 | 1:500 |
| ERK1 | 44 | Rabbit | Santa | Monoclonal | sc-271269 | 1:300 |

GAPDH = glyceraldehyde-3-phosphate dehydrogenase; ERK1 = extracellular regulated protein kinases 1.

(A) (B)

(C) (D)

(E)

**Fig. S1.** Effect of dietary Zn levels on egg weight (A), laying rate (B), egg production (C), feed intake (D) and feed/egg ratio (E). All values are expressed as means ± SE. MZD = maternal Zn-deficient group (0 mg Zn/kg diet); MZA = maternal Zn-adequate group (60 mg Zn/kg diet); MZH = maternal Zn-high group (120 mg Zn/kg diet).
